# The impact of prescribed psychotropics on youth

**DOI:** 10.1186/1745-0179-3-21

**Published:** 2007-10-20

**Authors:** Shaheen E Lakhan, Gareth E Hagger-Johnson

**Affiliations:** 1 Global Neuroscience Initiative Foundation, Los Angeles, CA, USA

## Abstract

Many psychotropics prescribed to children are unlicensed or off-label. This article uses the two most prescribed psychotropics (MPH and SSRIs) to illustrate various concerns about their impact on youth. Many mental illnesses begin in childhood or early adulthood, warranting a treatment of some kind. However, commentators have argued that prescribing is influenced by five myths: (1) children are little adults; (2) children have no reason to develop depression or anxiety; (3) psychiatric disorders are the same across adults and children; (3) children can be prescribed lower doses of the same drug; (5) drugs are preferable to alternative treatments and are more successful. Several lines of evidence suggest that these are incorrect assumptions. We update readers with recent research in relation to these myths, concluding that researchers should clarify child/adult differences for psychotropics, attend to the growth of "cosmetic" use of psychotropics in children and adolescents, and address concerns about the diagnostic validity of mental illness in the current DSM classification system.

## Background

At the level of individual use, both legal (alcohol, tobacco) and illegal (amphetamine, cannabis, and cocaine) psychotropics have a well-documented, short- and long-term impact on young people's health. Psychotropics include antidepressants, stimulants, antipsychotics, benzodiazepines, and other anxiolytics [1]. This article considers prescribed psychotropics.

Many psychotropics prescribed to children are unlicensed or off-label, meaning that they are used for purposes other than for which they are officially approved [2]. However, little is known about their long-term impact. The dramatic rise in prescriptions over the last ten years has raised concern about the potential for harm [3,4].

This article uses the two most prescribed psychotropics as examples to illustrate the concern about their impact. Methylphenidate (MPH, e.g., Ritalin) and selective serotonin reuptake inhibitors (SSRIs, e.g., Prozac, Seroxat) are prescribed most often [5]. Yet, very little research into their safety and efficacy with children and young people exists. Clinicians may therefore prescribe off-label in the absence of reliable data. Fortunately, clear reasons for the lack of data exist, and the situation is improving in several ways.

## Knowns and unknowns

Psychotropics can form an important part of treatment intervention for certain mental illnesses. Successful treatment of psychological disorders at a young age has the potential to reduce adult morbidity [6]. Many mental illnesses begin in childhood or early adulthood, warranting a treatment of some kind. Several psychotropics have proven effective. For example, stimulants can treat attention deficit hyperactivity disorder (ADHD). SSRIs are approved for treatment of obsessive-compulsive disorder [7], depression [8] and anxiety disorders in children. A recent clinical trial by the National Institute for Health and Clinical Excellence in the United Kingdom showed fluoxetine (with or without cognitive behavioral therapy, CBT) "to be the only selective serotonin reuptake inhibitor that is more effective than placebo in treating teenage patients with depression" [cited in 2, p. 1464]. These drugs may not be appropriate in all circumstances, however. The FDA approved Ritalin only for treatment of ADHD in children six years or higher [3]. Some antidepressants (sertraline and fluvoxamine) have been approved for OCD but not depression in school-age children [[5] , p. 983]. A warning was introduced by the FDA and the European Medicines Agency in 2003–2004 about the increased suicide risk of initiating SSRI treatment with antidepressants in children and adolescents, which has lowered the number of prescriptions made in recent years [9]. Gibbons et al. suggest that this may explain increasing suicide rates, because it results in untreated depression. This is clearly an important area for future research and "a major priority in designing national suicide prevention strategies" (p. 1361). Although a meta analysis showed no statistically significant differences between SSRI and control groups, [10], a more recent study found suicidal events occurred at a rate of 4.7% in the "SSRI plus CBT" condition, and 9.2% in "SSRI alone" condition (significant at p = 0.0402 vs. placebo), compared to 2.7% in the "placebo" condition [11]. However, an alternative explanation for the SSRI-suicide association is that patients "acquire energy to act on continued suicidality and/or may gain cognitive clarity to act on their suicidal intentions" [[12] , p. 84]. Joiner et al. argue that the increased "energy" after remission hypothesis is more likely due to incomplete remission in patients who were more depressed, from the outset.

## Recent trends

Despite the narrow range of situations in which prescriptions can be supported by safety and efficacy data, drugs are prescribed for an increasing range of disorders and for increasingly younger patients. We have seen a large increase in prescriptions for antipsychotic drug use, not accompanied by any parallel rise in the prevalence of psychotic illnesses [1,2]. The UK has had the highest percentage increase (68 percent), with Germany the lowest (13 percent) [1]. The ten-year prevalence of MPH prescriptions increased by up to 3.7 times for Medicaid patients and 7.2 times for HMO patients [Safer, Zito & Fine, 1996, cited in 3]. In the UK, MPH prescriptions increase from 2000 in 1990 to 350 000 in 2004 [[13], p. 64]. Trends by age have also changed, with 10- to 14-year-olds now most often treated with stimulants. This could suggest "a lengthening of the duration of treatment" [2]. For SSRIs, gendered patterns of prescribing have also changed. Historically, males have tended to receive more stimulants, "but the sex gap has narrowed, and SSRI prevalence by sex was approximately equal between males and females in 1998" [[14], p. 564]. None of these trends parallel equivalent changes in the prevalence of the mental disorders the psychotropics are designed to treat, which suggests a discrepancy between prescribing practice and disorder prevalence [see [14-16]].

Up to 60 percent of drug prescriptions in the U.S. are off-label, a sizeable portion of which are written for children and adolescents [17]. The principal reason why prescriptions are written off-label is the lack of research (discussed below). Clinicians are justified in prescribing off-label because they have to balance *beneficence *(using professional judgement to treat and enhance quality of life) with *nonmaleficence *(protecting from unsafe or ineffective treatments). In the absence of research into psychotropics for children and young people, little choice may exist, other than to use treatments with proven effectiveness for adults. Wiznitzer & Findling [18] discussed five popular myths in relation to psychotropics and youth. Here, we use these myths to update readers with recent research findings, debates and practice guidelines.

### 1. Children are little adults

The putative biological substrate for psychiatric disorders might vary as a function of age. The therapeutic effect, potential adverse events, and interactions with other drugs, can therefore vary with age. The brain evolves continues to evolve throughout adolescence [18], providing what might be termed a *moving *target for clinicians and researchers. The underlying biological substrate for depression, for example, might change, even if the psychological construct that defines depression does not [19] and "there is inadequate research on the neurodevelopmental consequences of recurrent or chronic episodes of mood disorder during different periods of brain development" at the clinical level [[19] , p.964]. This may explain "the lack of efficacy and poor side-effect profile for tricyclic antidepressants" [[20], p. 1341]. These older drugs have long been discouraged for use on the young. More recently, SSRIs discouraged for use as treatments for depressed children and adolescents [20]. This change was informed partly by the observation of increased suicide risk among adolescents taking SSRIs, discussed in more detail below [9,10,21]. Leeder argues that "infants, children, and adolescents simply cannot be treated as 'small adults.' Rather, they represent distinct subpopulations, each with their own unique characteristics, and, thus, the response to drug therapy from birth to adolescence and adulthood is a function of developmental processes that affect drug disposition and the interaction of drugs with their pharmacologic targets" [[22], p. 765].

The first myth, that children are little adults, is particularly important, given recent research into the developing brain. The developing brain is anatomically vulnerable. The significant developmental differences between adults and children have implications for pharmacological effects [23]. As the brain develops, cellular events are "programmed" to unfold in a particular sequence. Disturbance of this normal process of self-assembly is a central mechanism in many disease processes. Neurons play a different role in the developing brain than in the adult brain. Carrey [23] argues this could affect response to psychopharmacological interventions. Long-term damage is possible, and psychoactive drugs could interact with brain development. The long-term safety of psychotropic agents for the brain is not known, and safety/efficacy studies for preschoolers are few and far between [5,24]. It is possible that stimulants such as Ritalin can desensitize the brain, leading to later substance abuse [5]. Phenobarbital can cause permanent negative effects on cognition [17]. SSRIs have been linked to irritability, shivering, agitation, hypotonia, and pulmonary hypertension in newborns [17,25].

Why is there so little research into the safety and efficacy of psychotropics for children and young people? According to Spetie & Arnold [17], although controlled trials have been conducted for psychiatric disorders in children [for examples, see [4]], these were historically more difficult to conduct. Children are defined as a vulnerable population, and it can be difficult to obtain informed consent from minors or their parents. Researchers have therefore been historically reluctant to enrol children in psychopharmacological research, "leading to a scarcity of data on the effectiveness, safety and pharmacokinetics of psychoactive agents" for preschoolers [[17], p. 16]. This reluctance has arguably made children *more *vulnerable, because little safety data exists for clinicians to refer to. Clinicians may not see it as appropriate to withhold a potentially useful treatment simply because it has not been approved for children. This situation is changing [see [4]]:

• From 1994, the FDA has required pharmaceutical companies to test certain drugs at pre-market stage for children. Prior to this, drugs were simply labelled with "safety and efficacy not established for children." There now exists a financial incentive to conduct pediatric studies, such as the six-month patent extension for fluoxetine [see [17]].

• From 1996, Pediatric Pharmacology Research Units (PPRUs) set up by the National Institute of Child Health and Human Development have helped limited efficacy and pharmacokinetic studies take place [4].

• From 1997, the Research Units on Pediatric Psychopharmacology (RUPPs) produced pediatric data on the efficacy of several psychotropics, funded my the National Institute of Mental Health (NIMH).

• From 1997, pharmaceutical companies were granted a "6-month extension of drug exclusivity...in return for specific pediatric studies" by the US congress [see [4]].

• In 2002, this was extended to 2008 under the Best Pharmaceuticals for Children [4].

• In 2003, pediatric studies were now required "as part of the premarketing development of drugs" [[4], p. 6].

There does, however, remain a set of factors which make ethical approval more difficult to obtain. For example, informed consent is more difficult to achieve in the presence of cognitive immaturity, parents' protective roles may be influenced by the emotional state of the child, coercion may exist from parents or indirectly via high financial reimbursements, placebos (discussed below) may need to be administered "to determine effective treatment when no or poor evidence base" [[17], p. 16].

A second reason why there is comparatively little research is that it is difficult to obtain ethical approval specifically to study the *mechanistic *effects of drugs. Ethical approval is easier to obtain if the study offers children a direct benefit [24]. Mechanistic studies are highly informative, but do not necessarily offer the individual patient any direct benefit [24]. Third, and related, is that children may be assigned to a dummy treatment group where no real medication is provided. This improves the reliability of clinical trials because placebo effects are known to be quite strong [17]. A placebo effect occurs "When the effects of a drug are temporally correlated with its administration and cannot be attributed to its chemical properties – its pharmacodynamic activity – they are known as placebo effects" [[26], p. 282]. Patients, families, and clinicians can all believe that a patient's condition is improving when, in fact, no medication was administered. This is why the "best" clinical trials are deemed to be randomized, placebo-controlled trials. Researchers disagree on whether a placebo condition offers children any direct benefit, because the findings are mixed, depend on the condition, and raise ethical concerns [7,10,24,27]. However, they generally recognize that placebo effects should be included in research designs [28]. On the one hand, they still receive a "non-specific, repeated clinical contact" which has demonstrated benefits [[24], p. 89]. On the other hand, they are not receiving any potentially helpful psychotropic medication. Vitiello [24] argues that placebo conditions are more justifiable for children, because there is so little safety/efficacy research available. Sometimes the placebo effect is so strong it cannot be considered a true absence of treatment [see [28]]. When the efficacy of a drug is known or demonstrated, the situation is more complicated. Many consider it unethical to withhold treatment from a group of children when we know the treatment works. Spetie & Arnold [17] cite "the notorious case of the Tuskegee study, in which syphilitic patients were left untreated, even after the discovery of penicillin" [[17], p. 22]. It is considered ethical to stop placebo-controlled trials if a strong effect of the drug becomes apparent [29].

### 2. Children have no reason to develop depression or anxiety

The beliefs that mood disorders are rare before adulthood and mood disturbance is a normal aspect of child and adolescent development are misconceptions [30]. There is a large and growing literature demonstrating the incidence and prevalence of pediatric depression, anxiety, and other mood disorders (e.g. mania, obsessive compulsive disorder). Reviewing these rates, Hankin, Abramson, and Siler [31] report that between 15 and 18 years of age, one-year prevalence of depression increases from 3 percent to 18 percent. Depressed mood is reported by 25 to 40 percent of adolescents, and researchers estimate "that 2–6% of children and adolescents in the community suffer from depression [20,32], p. 1341]." This implies the existence of a critical period during which the risk of depression is highest. Children are surrounded by life stressors, and may not develop adequate coping mechanisms [18]. Moreover, the number of negative life events experienced increases during middle to late adolescence. This may offer a partial "explanation of why so many individuals become depressed for the first time in adolescence [[31], p. 624–625]." Evidence that self-reported depression corresponds with parent and teacher ratings provides validity for the existence of this disorder in youth, despite its "internalizing" nature [see [33]]. It is not widely agreed, however, how data from multiple informants "should be combined to yield diagnoses [[30], p. 1003]." Some of the consequences of depressive disorders may reflect not only impairment of the disorder, but may represent a core symptom of the disorder itself. The phenomenology of depressive disorders in children may change as a function of age, which is discussed below.

## Risk factors for child and adolescent depression

Children can develop mood disorders, and there are risk factors. For example, negative cognitions (thought processes) about the self are predictive of depression in adults and children [34]. Low self-esteem, negative self schemas (representations of the self) and/or negative cognitions "are hypothesized to play a role in the onset and maintenance of depression" [[34], p. 371]. Schemas are "enduring mental constructs [that] organize, guide, interpret, and retrieve information about the self in memory [[35], p. 216]." Depression may occur when the self is diminished in some way, by "a loss, failure, depletion, evidence of undesirability or inefficacy [[36], p. 218]."

Perceived control over life events is a second risk factor for depression. Although only limited research is available, studies to date suggest children with an external locus of control, or a general perception of low control, are more likely to develop depression [34]. In the authors' own study, having a low academic grade interacted with the presence of a generally negative attributional style; to produce negative affect and depressive symptoms for preadolescents. Some of these symptoms occurred before the stressor itself, suggesting children anticipated the stressor or a general tendency to experience these symptoms existed. Those without negative cognitions were less likely to develop depressive symptoms after receiving a lower than expected grade. Students with positive cognitions were protected against depressive symptoms, even among those receiving lower than acceptable grades. These results show that stressful life events in children can lead to depression, particularly when moderated by negative cognitions. Other researchers argue, however, that life events are a stronger predictor than the "hopelessness as mediator" hypothesis. These events are stressors, which can provoke learned helplessness and hopelessness in children. Learned hopelessness refers to "the expectation that highly desirable outcomes will not occur or that highly aversive events will occur [[31], p. 608]." Depression is more likely among those with stable, global and internal cognitive processes ("negative beliefs regarding themselves, their future, or their situation [[31], p. 378]." Not all individuals react in this negative way, suggesting that "negative cognitions are a vulnerability factor that interacts with negative life events to contribute to the onset and maintenance of depression [[34], p. 370]." In the revised version of the learned hopeless theory, the stable and global aspects of these negative cognitions considered to be more important than the internal dimension [31] -negative beliefs that the event will always happen and will happen in most situations. An empirical test of negative cognitions versus life events in the onset of adolescent depression found more support for stressful life events. The importance of life events remained, regardless of whether "stable + global + internal" or simply "stable + global" styles were considered. The increase in stressful life events during adolescence may correspond with and partially explain the increase in the prevalence of depression.

The heritability of bipolar disorder is particularly well-documented [30], with strong evidence for genetic contributions to depression and other disorders. It is plausible that reducing the incidence of depression in children and adolescents "might be successful in preventing subsequent adverse role transitions and, indirectly through this effect, might reduce the prevalence of mood disorders in adulthood [[30], p. 1010]." Genetic factors are key determinants of mood disorders, making it likely that the onset and course of the disorder can be identified earlier than in adulthood. However, researchers will need to consider gene-environment interactions. The influence of parental disorders on child disorders could be genetic, environmental (e.g., parenting style), or both. The pathways are difficult to discern because parental disorders are "part of a complex cluster of risk factors [[30], p. 1007]." Parents' behavior can create environments in which stressful life events occur, making it difficult to disentangle the genetic contribution from the environmental contribution.

### 3. Psychiatric disorders are the same across adults and children

Although the phenomenology of depression appears to be similar in adolescents when compared to adults, key differences in symptomatology exist [37]. The clinical picture varies as a function of age. Younger children tend to show "anxiety, somatic complaints, auditory hallucinations, and behavioral problems", where older children and adolescents tend to show "sleep and appetite changes, suicidal ideation, and impairments of functioning" [[37], p. 8]. Features that would be considered consequences of depression in older children and young adults (e.g. declining school performance), might be considered a symptom of the disorder itself, in younger children [37]. Kessler et al. [30] note the "important exception is that irritability is included as a core symptom of depression among children and adolescents, but not adults [[16], p. 1003]." They suggest that if irritability were included as a component of depression for males, this might have the additional side benefit of reducing gender differences in rates of depression. Put differently, prevalence may be the same, but men are more likely to present irritability as a manifestation of their depression. Asecond way in which disorders may differ between adults and children is the extent to which symptom profiles should be considered "clinically significant." Many studies apply the same diagnostic thresholds used for adults onto children. Thresholds are designed to "employ explicit rules for assessing syndromes in terms of frequency, duration, and impairment [[30], p. 1003]." Yet there are "intriguing differences in depression symptom profiles with age [[30], p. 1003].

 Older children show decreases in self-esteem, somatic symptoms, and agitation. Mania is a good example of how diagnostic thresholds may need to adapt to different age groups. A large proportion of children (5 to 11 percent) show manic-like symptoms, but because these persist only for a few hours or days, they do not meet the formal, adult threshold for bipolar disorder. Euphoria and irritability would need to persist for four or seven days, for bipolar II and I, respectively. Furthermore, mood disorders are often comorbid with other disorders, such as anxiety, ADHD, or substance use [38]. The extent of comorbidity may be different in adolescents than in adults. Issues surrounding diagnosis may reflect problems with the diagnosis of mood disorders themselves, rather than adult/child differences. The Diagnostic and Statistical Manual of Mental Disorders (DSM) [39] places mood disorders and substance use disorders on Axis I, but there is considerable overlap with the personality disorders of Axis II (discussed below). Furthermore, "DSM criteria are not sufficiently explicit to provide clear guidelines regarding a number of required classification distinctions, such as "clinically significant" impairment and "marked" distress" [Angold et al. 1995, cited in 30, p. 1004]. Many patients receive more than one disorder, leading to concern about the validity of categorical approaches to mood and personality disorders [38]. Kessler et al. [30] note that children and adolescents with bipolar disorder almost always have at least one other disorder. They argue that an "admixture of manic, attention deficit, and hyperactive symptoms [[30], p. 1007]" may in fact reflect diagnostic confusion. The fact that the phenomenology of disorders may change as a function of age, complicates DSM and other classificatory schemes further. The definition, onset, duration and impairment caused by the disorders may have to be qualified with age-specific information, to reach the *moving target *that constitutes the child's disorder. Measures designed to diagnose disorders would have to take this into account, rather than simply adapting measures designed for adults. The point that children are not small adults (discussed above) applies to diagnosis, as well as biological substrates. Ideally, age-sensitive measures would be adaptable for use in primary care settings. Primary care is where most depression is diagnosed and treated, and is the first point of contact for children with disorders [37].

Adults and children also differ in the extent to which their disorders are treated. In their review, Kessler et al. [30] note that (1) the vast majority of depressed adolescents seek help, (2) delays in help seeking are related to age, and (3) the likelihood of receiving help has increased in recent years. Research into patterns of help seeking is particularly informative, because the consequences of untreated depression are dangerous for young people. A "pileup of cumulative adversity [[30], p. 1009]" from early mood disorders can increase the likelihood of unfavorable social outcomes, such as low education attainment, teenage pregnancy, and secondary substance abuse. Unfortunately, these outcomes may occur before help seeking takes place. Externalizing symptoms, such as mania, are easier for clinicians to identify, making internalizing disorders more problematic. Early identification and intervention may prevent future psychopathology. Kessler et al. [30] note that, "Given that early-onset depression is often preceded by one or more anxiety disorders, interventions aimed at childhood-onset primary anxiety might be even more effective [[30], p. 1010]." Clearly, longitudinal research into the symptoms, diagnostic validity, and treatment options for children and adolescents would be particularly valuable. Despite a paucity of research, sufficient evidence indicates that psychological disorders are not the same for adults and children.

### 4. Children can be prescribed a lower dose of the same drug

Evidence to support the claim that children can be prescribed the same drugs as adults but at a lower dose is limited. Wiznitzer & Findling [18] note that pharmacokinetic, pharmacodynamic, and response effects change across different age ranges. Factors such as metabolic rate can be taken into consideration when administering doses of psychotropics, so that doses can be adjusted depending on body weight. Age alone is not an appropriate variable on which to adjust dosage. Age "is the more dominant factor in the variability of drug action in the infant and young child" but later in life, age and body size diverge and dosage may need to be tailored to weight [[26], p. 260]. Dosage options should be informed by "scientifically-based investigation rather than weight adjusted dosing extrapolated from adult data [[18], p. 1147]." Several side effects from SSRI use in children have been described in the literature, including insomnia, fatigue, abdominal discomfort, sleep problems, decreased appetite, and excitement see [40]. Some of these effects are temporary, and may depend on the length of treatment, but "the long-term effects of these drugs on the developing nervous system is not known and needs to be weighed against the potentially serious and noxious consequences of no treatment and persistent symptoms [40]." Weintrob et al. describe four children (aged 11 to 14) who showed decreased growth during SSRI treatment. They attributed this to a decrease in growth hormone secretion via "a selective impairment of the somatotrophic axis by SSRIs [[40], p. 699]." The authors called for more research and warned clinicians to be wary of the potential for restricted growth, given the increasing use of SSRIs with children. Eventually, pharmacogenetics, "the increasingly important branch of pharmacology that deals with the study of genetic modifications of drug response [[26], p.266]," will need to be considered in relation to pediatric psychopharmacology. The field concerns individual differences in drug response, and the study of "changes in phenotype that occur as a child grows and Develops". This is an emerging discipline: "pediatric or developmental pharmacogenetics is essentially at a neonatal stage [[22], p.777] [see also [4,41]]."

### 5. Drugs are successful at treating psychiatric disorders

Psychotropics are only one of several treatments available for psychiatric disorders in children and adolescents. Obsessive-compulsive disorder (OCD) is a useful example of a disorder usually treated with CBT and psychotropics, rather than psychotropics alone. OCD involves compulsive behavior, such as "checking, washing, repeating, touching and straightening" [[42], p. 51]. Although the phenomenology of OCD is the same, differences exist in children and adolescents when compared to adults on the gender distribution, comorbidity, genetic contribution, and development of OCD [42]. Prevalence estimates vary from 0.2 percent to 1.2 percent in clinic settings to 1 to 4 percent in community settings. Shafran notes it is difficult for young people to verbalize their compulsions; they "will often say that the compulsion is driven by a sense that 'it doesn't feel right' unless such compulsive behavior has been completed [[42], p. 51]." Since OCD is comorbid with other disorders, diagnosis of OCD in children is difficult. OCD can be confused with anxiety disorders, again highlighting the importance of diagnostic precision in DSM. There lacks a reliable and valid "gold standard" assessment instrument, although semi-structured interviews are available.

In CBT, the goal is usually to expose the child to the feared stimulus gradually, which blocks the negative reinforcement effect of the compulsive behaviors (called exposure and response prevention, or E/RP). CBT can help the "patient make rapid and difficult behavior changes over short time intervals [[6], p.16]." The cognitive component of the therapy involves the development of a psychological "tool kit" to help improve their "sense of personal efficacy, predictability, controllability, and self-attributed likelihood of a positive outcome with E/RP tasks" [[6], p. 9]. The compulsive behaviors can be terminated through a process of extinction (by removing positive reinforcement of the behaviors) and modeling:

"Modeling–whether overt (the child understands that the therapist is demonstrating more appropriate or adaptive coping behaviors) or covert (the therapist informally models a behavior)– may help improve compliance with in-session E/RP and generalization to between-session E/RP homework [[7], p. 10]."

In contrast to depression, CBT alone, especially for younger patients, or "CBT + medication" is the standard initial treatment [see [43]]. CBT is an empirically supported treatment, which March et al. [6] argue has proven more successful than insight-oriented psychotherapy. The emphasis on CBT probably reflects the cognitive basis of the disorder. OCD is characterized by intrusive thoughts, which the patient appraises "as indicating responsibility for harm or its prevention" [[42], p. 52]. This responsibility drives patients to take "corrective action" in the form of the repetitive behaviors. A large literature supports the efficacy of CBT for treatment of OCD in adults, and a smaller literature supports it for children. SSRIs have also shown effectiveness and are permitted for use in children (unlike for depression) [42]. In a randomized, controlled trial comparing CBT with clomipramine, the mean improvement was higher at 60 percent in the CBT group than in the clomipramine group at 33 percent [44]. Alternative treatments can therefore be used successfully alongside psychotropics. Some argue CBT combined with SSRIs is the most optimal strategy [6,27]. However, a recent British trial found that "the addition of CBT adds little to specialist active clinical care in conjunction with an SSRI in the short term", at 28 weeks, despite the fact that CBT is recommended as a treatment of choice by the National Institute for Health and Clinical Excellence (NICE) [45]. In primary care settings, medication will usually be favored and detract from nonpharmacological interventions [17,45]. Parent training is another example of a nonpharmacological intervention that can help treat and prevent conduct disorders [46]. It is interesting that many nonpharmacologic treatments are not covered by insurance–even when they are cheaper and would perhaps be more effective than psychotropics. The dramatic rise in prescriptions has alarmed several commentators [notably [1,18]]. In reality, the prescription of psychotropic drugs is multi-determined. Prescription trends are influenced by societal factors and pressures: regulatory guidelines and the law, local policies, professional guidelines, clinicians' own theoretical orientation, culture, parents, family values, schools, and the media [1-4,13,17,43,47,48]. In the next section, we use the example of ADHD to illustrate how multiple, social factors can influence psychotropic prescription trends.

## Validity of ADHD as a diagnosis

Another set of concerns about the prescribing of psychotropics in children and young people is about the validity of the diagnosis itself. Although ADHD, oppositional defiant disorder, and conduct disorder have been validated, ADHD remains a controversial disorder. Concern exists that "elevated but still developmentally normal levels of motor activity, impulsiveness, or inattention" traits of childhood could be inappropriately interpreted as ADHD [5]. ADHD symptoms could mask other disorders, such as mood disorders or cognitive impairments. Some argue that Ritalin is prescribed for cosmetic reasons. Cosmetic psychopharmacology refers to "pharmacologically improving the brain functioning of healthy, normal individuals [[49], p. 113]." Social pressure means that "passing exams becomes ever more important to gain a satisfactory job, and pass rates in school exit exams continue to rise year on year [[1], p. 1465]." MPH will maintain self-administration in laboratory animals, because it increases dopaminergic activity in the same reward pathway as cocaine and amphetamine. Children may be encouraged to buy or sell Ritalin. Adolescents with ADHD are already more likely to abuse other substances (e.g., nicotine), making them a high risk group because "college years are a time of psychoactive drug use [[50], p. 142]." In their sample, Babcock and Byrne [50] found that 16.6 percent of subjects reported taking Ritalin for fun, 16 percent had snorted the drug, and 35.7 percent knew students at the college from whom they could buy Ritalin [see also [13]]. Students may be "pulling all nighters," indicating a form of self-administered cosmetic psychopharmacology (enhancement of brain functioning without medical need). The authors concluded the abuse potential of MPH should be considered when prescriptions are made and MPH abuse "must be viewed as a public health problem [[50] , p. 145]." Parents, wanting to give their child the best start in life, report they feel justified in treating their children with Ritalin [48]. Indeed, the number of prescriptions written for Ritalin far outweighs the estimated prevalence of ADHD, suggesting that cosmetic use is occurring [4]. This does not mean that cosmetic use exists purely beyond full treatment of children with ADHD. Under-treatment of ADHD can occur at the same time as over-treatment, "as some children with ADHD may not be adequately treated while others who do not have ADHD are treated [[4], p.6] [[13], p. 64]." Indeed, there is a possibility that "unmedicated children or adolescents with ADHD may be more likely to "self-medicate" by seeking out alcohol or illegal psychotropic agents [[13], p. 67]." Mood disorders in younger children, particularly younger than six [2]. Treatment is usually focused on controlling symptoms, rather than declaring a categorical mood disorder, such as depression.

## Addressing the absence of research

To address shortcomings in the research literature, the National Institute of Mental Health funded a multi-site trial, the Preschoolers with ADHD Treatment Study (PATS). Given the 1.7 to 3.1-fold increase in MPH use between 1991 and 1995 reported by Zito et al. [3], the PATS study was launched in 2001 to address growing safety and efficacy concerns. PATS took place over six sites. Using strict criteria, eligible children shown to have ADHD and able to tolerate treatment were randomized to one of five conditions that differed by dose and, crucially, a placebo condition. Following the study, all children were eligible for ten months of treatment. This addressed ethical concerns about withholding potentially beneficial treatment, while also providing "systematic information regarding the safety and relative long-term effectiveness of MPH [[51], p. 1279]." The primary outcome measure of interest was changes on parent and teacher rating scales–validated and clearly defined measures of ADHD-related behavioral functioning. The study was one of the first to show an advantage of low MPH doses over placebo in the classroom setting [47]. The dose-response relationship was smaller in school-age children on the same medication, showing that differences do indeed exist between pre-schoolers and school-age children which clinicians need to consider. The study also showed the existence of some side effects, however. Compared to the placebo condition, MPH-treated children were more likely to show decreased appetite and trouble sleeping throughout the study, as well as weight loss at the beginning of the study. In total, eleven percent of children enrolled in the study had their participation ended because of intolerable adverse events [52,53]. Swanson et al. found that treatment slowed children's growth rate about 20 percent less than expected for their height, with reductions of around 50 percent for weight after one year of continuous treatment. Although these observations need to be balanced against the risks of untreated ADHD, Wigal et al. concluded "those preschoolers with ADHD on MPH treatment still need to be carefully monitored [[53], p. 1302]." The PATS study is a welcome addition to the literature on psychotropic treatment of youth.

## Three research priorities for the next decade

Three areas of research are likely to feature prominently over the next decade. The first is the relationship between psychotropics and stress. A complex relationship exists among depression, stress, and stress hormones. The hypothalamic-pituitary-adrenal axis (HPA axis) regulates production of the stress hormone cortisol. In depression, levels of cortisol are markedly elevated, and this may lead to hippocampal damage. It is interesting that stress can inhibit hippocampal neurogenesis, but SSRIs can induce neurogenesis [19]. In rats, dentate granule cells in the hippocampus increase by 70 percent after three weeks of fluoxetine. Production of these cells can be suppressed by stress or cortisol. Longitudinal studies of cortisol and hippocampal volumes, comparing children treated with SSRIs and controls, will be particularly informative. The importance of life events as triggers of depression underscores the need to consider stress in more detail [31].

Second, a need exists for more research into the cosmetic use of psychotropics by young people or parents of younger children. As mentioned above, parents may seek to capitalize on the side effects of psychotropics for improving the educational attainment of their children [48]. At the extreme, reports exist of Ritalin abuse by adolescents who want to experience increased levels of concentration or the "high" associated with the drug [50]. Although Ritalin is similar to cocaine in terms of its dopamine release mechanism, the clearance rate is much slower, which may explain why it is not abused more [see, [23]]. Sutcliffe and Wong [1] argue that practitioners need to remain wary of both industry and societal pressures to prescribe psychotropics when other options are available.

Finally, research into the diagnostic validity of psychological disorders in children is likely to continue. The forthcoming DSM-V will attempt to address the problems associated with construct overlap, multiple diagnoses, and overlapping symptoms, which have hampered attempts to adequately describe and treat specific disorders. Comorbidity of mood, substance, and personality disorders may reflect a real tendency for children's symptoms to co-occur or to reflect the limitations of the current scheme to differentiate adequately among distinct disorders. An alternative scheme has been proposed which addresses this problem:

"The factor connecting the unipolar mood and anxiety disorders was labeled *internalization*, to describe the propensity to express distress inwards that unites these disorders. The factor connecting the substance use and antisocial behavior disorders was labeled *externalization*, to describe the propensity to express distress outwards that unites these disorders. Importantly, internalization and externalization are conceived as separate dimensions, as opposed to opposite ends of the same dimension [[54], p. 1248]."

The tendency of "internalizing" and "externalizing" disorders to co-vary is worth studying in its own right, rather than being treated as a diagnostic problem *per se *[38,54]. According to Krueger et al. [[54], p. 1257], "SSRIs may be better viewed as treatments for the core, shared temperamental component of the internalizing disorders". Comorbidity is the rule, rather than the exception, in child and adolescent psychiatric disorders. For example, ADHD frequently overlaps with internalizing and externalizing disorders [55]. Attempting to study disorders in isolation by conducting research on "pure" cases could limit the applied benefits of research. Efficacy trials are usually conducted on healthy adults of a limited age range. The typical "linear model" (from efficacy studies to effectiveness studies, dissemination, and implementation) may not be appropriate. In real-world clinical settings, patients may have multiple problems, which brings "a large array of new, confounding variables [[19], p. 967]." Hyman argues that the exclusion criteria for many studies mean we have limited information on young people most at risk. Studies usually exclude patients at risk of suicide, meaning little information exists on how to manage and treat suicidal adolescent patients.

## Conclusion

We can expect to see the development of child and adolescent versions of questionnaires and interviews, the development of normative data for childhood mental disorders, and safety/efficacy studies. These studies will need to be conducted on specific age groups, specific disorders, and with specific treatments. Prescribed psychotropic medications are now high on the research agenda. Recent news coverage, as well as changes in both clinical practice standards and drug product labelling, have extended researchers' concerns into the wider public [see [3]]. The traditional reluctance to enrol children in clinical trials of psychotropics, such as MPH or SSRIs, has had the effect of increasing, not decreasing, their vulnerability as a special population. Continued discussion about the ethical challenges of research [17,24,28,48,49] and the development of well-designed, placebo-controlled trials, have begun to change this situation. For the first time, we can begin to record and measure, rather than assume, the impact of prescribed psychotropics on children and adolescents. Few would disagree with Spetie and Arnold [[17], p. 21] that "children and adolescents have the right to treatment based on accurate, age-appropriate data."
